# COVID-19 (SARS-CoV-2-Infektion): Einfluss auf die männliche Fertilität

**DOI:** 10.1007/s41973-023-00217-1

**Published:** 2023-06-07

**Authors:** Alexander Müller

**Affiliations:** 1 Uroviva – Klinik & Praxis Bülach, Zürichstr. 5, 8180 Bülach, Schweiz; 2 Universität Zürich, Zürich, Schweiz

**Keywords:** COVID-19-Infektion, Männliche Sterblichkeitsrate, Hypogonadismus, Erektile Dysfunktion, MRNA-Vakzinierung, Infection COVID-19, Taux de mortalité hommes, Hypogonadisme, Dysfonction érectile, Vaccins à ARNm

## Abstract

Höhere Sterblichkeit und ein schwererer Krankheitsverlauf sind bei Männern mit SARS-CoV-2-Infektion gegenüber Frauen zu beobachten. Die Hodenfunktion (Samenqualität und Testosteronhaushalt) sowie die Erektionsfunktion sind, zumindest kurzfristig, durch eine COVID-19 Infektion negativ gestört – wobei mögliche Langzeiteffekte noch nicht hinreichend geklärt sind. Bei einer angestrebten Fertilität, inklusive assistierter reproduktionsendokrinologischer Massnahmen oder Kryopräservation benötigen Männer u. a. aufgrund potenziell infektbedingter DNA-Schäden im Erbgut eine kompetente Beratung und einen engen Follow-up.

## Résumé

Une mortalité plus élevée et une évolution plus grave de la maladie sont observées chez les hommes infectés par le COVID-19 par rapport aux femmes. La fonction testiculaire (qualité du sperme et équilibre de la testostérone) ainsi que la fonction érectile sont négativement perturbées par une infection au COVID-19, du moins à court terme, bien que les éventuels effets à long terme ne soient pas encore suffisamment élucidés. En cas dʼobjectif de fertilité, y compris les mesures dʼendocrinologie de la reproduction assistée ou la cryopréservation, les hommes ont besoin dʼun conseil compétent et dʼun suivi rapproché, notamment en raison des lésions de lʼADN dans le patrimoine génétique potentiellement liées à lʼinfection.

## Einleitung

Bereits im Jahr 2003 lernte die Welt das Coronavirus 1 (CoV-1) kennen, welches damals lediglich für etwas mehr als 8000 Infektionen verantwortlich war. Es besteht zum CoV‑2 eine 79,6%ige genetische Ähnlichkeit, wobei diese Virusvariante ein viel grösseres Ausmass erreichte [1]. Im Dezember 2019 wurde in Wuhan, China, erstmals über eine Coronaviruserkrankung (COVID-19) berichtet, welche durch das „severe acute respiratory syndrome coronavirus 2“ (SARS-CoV-2) verursacht wird. In der Folge kam es dann zu einer weltweiten, pandemischen Ausbreitung. Obwohl es in Bezug auf die prozentuale Infektionsrate bei Männern und Frauen praktisch keinen Unterschied gibt, zeigte die statistische Analyse in allen Ländern für Männer einen schwereren Krankheitsverlauf und eine höhere Mortalitätsrate gegenüber Frauen [2]. Gründe hierfür bestehen u. a. darin, dass Männer einen ungesünderen Lebensstil pflegen (Rauchen, Übergewicht, Inaktivität) und somit mehr ungünstige Komorbiditäten aufweisen, aber auch in einem höheren Vorliegen von Androgenrezeptoren, was ein postuliertes Eintreten des Virus in die Zelle erleichtert [3].

## Viruseintritt

Das Coronavirus SARS-CoV‑2 ist ein Einzelstrang-RNA-Virus, welcher primär über Aerosole, ausgeatmet von infizierten Personen (geimpft und ungeimpft), oder auch Konjunktivalflüssigkeit und Tränen übertragen wird. Es konnte sowohl in Speichelflüssigkeit als auch im Urin, Fäzes und Blut nachgewiesen werden. Der Nachweis in der Samenflüssigkeit wurde in der Mehrzahl der Literaturangaben verneint [4] Somit scheint eine sexuelle Transmission eher unwahrscheinlich, kann aber nicht mit letzter Sicherheit ausgeschlossen werden. Vereinfacht dargestellt gelangt das SARS-CoV-2-Virus durch Bindung an sog. Spikeproteine am Angiotensinkonversionsenzym 2 (ACE-2), welches durch eine transmembranöse Serinprotease Typ II gekoppelt ist, in die Zelle [4, 5]. Eine besonders hohe Expression von ACE‑2 konnte in Lunge, Schilddrüse, Brust, Niere, Intestinum und auch im Hoden nachgewiesen werden [6, 7]. Durch eine infektionsbedingte Störung der Blut-Hoden-Schranke kann das Virus direkt Störungen sowohl der exokrinen (Testosteron) als auch der endokrinen (Spermatogenese) Hodenfunktion verursachen. Innerhalb des Hodens konnten wiederum hohe ACE-2-Expressonen sowohl in Leydig-Zellen (Testosteronproduktion) als auch in den Sertoli-Zellen, Spermatogonien und Spermatiden nachgewiesen werden [8]. Zudem besteht eine starke Korrelation zwischen der *TMPRESS2*-Expression und einem schweren Krankheitsverlauf [9]. Der Androgenrezeptor beeinflusst die Expression von *TMPRSS2* (höher bei Männern als bei Frauen). Je höher die *TMPRSS2*-Expression, desto schwerer kann der Krankheitsverlauf ausfallen. Für die potenzielle COVID-19-verursachte Hodenfunktionsstörung bedeutet dies, je jünger die Männer sind, desto höher ist die ACE-2-Rezeptor-Expression und somit der zu erwartende testikuläre Schaden [10]. Dies wird u. a. durch das Generieren von oxidativem Stress in Form von sog. „reactive oxygen species“ (ROS) verursacht. In der Literatur sind 10–22 % Entzündungen des Hodens (Orchitis oder Epididymoorchitis) bei einer akuten COVID-19-Infektion beschrieben [11].

## Einfluss auf die männliche Fertilität

Neben einer direkten viralen Schädigung wird bei einer akuten COVID-19-Infektion bis zu 98 % Fieber als Symptom beschrieben. Eine erhöhte Körpertemperatur betrifft auch den Hodenbereich mit potenziell schädigendem Einfluss auf die vulnerable Spermatogenese. Kritisch scheint dabei eine Temperatur von mehr als 38 °C über mehr als 2 Tage im Hodengewebe zu sein, was dann zu einer konsekutiven Verminderung der Spermienkonzentration, Vitalität, Motilität und Morphologie bei erhöhter DNA-Fragmentation führt [12].

Die aktuell stetig wachsende Literatur hat aber aufgrund von eher kleinen Fallzahlen, unterschiedlicher individueller Krankheitsausprägungen und v. a. durch fehlende Vergleichsdaten vor und nach Infektion (noch) gewisse Limitationen.

Durch eine COVID-19-Infektion konnten multiple negative Einflüsse auf die Samenqualität nachgewiesen werden. Dabei wurden eine Verminderung der Samenkonzentration, der Beweglichkeit, der Vitalität und der Morphologie bei gleichzeitigem Anstieg des ROS und der DNA-Fragmentation beschrieben [13]. In einer frühen prospektiven Querschnittsstudie zeigte sich, obwohl kein Nachweis von Virus-RNA in der Samenflüssigkeit bestand, in 73 % eine schwere Spermatogenesestörung (Krypto‑/Azoospermie) bei Männern, die bereits Vater geworden waren [14]. Der Schweregrad der Spermatogenesestörung korrelierte dabei stark mit der Schwere des Krankheitsverlaufs (z. B. Notwendigkeit zur Hospitalisierung). In einem systemischen Review der Literatur konnte bei Männern mit COVID-19 im Vergleich zu einer gesunden Kontrollgruppe eine signifikante Reduktion der Gesamtspermienzahl, der Spermienkonzentration, der Motilität und des Samenvolumens gezeigt werden [15]. Dies wurde auch in einer Metaanalyse im Jahr 2022, die 7 Fall-Kontroll-Studien und 5 retrospektive Kohortenstudien einschloss, bestätigt werden und somit betätigen, dass eine solche Infektion eine deutliche Vulnerabilität bezüglich der Samenqualität bewirkt [16].

Hingegen besteht nur bedingt Grund zur Sorge, da mehrfach gezeigt werden konnte, dass nach 3–4 Monaten (rund 3 Monate entsprechen einem Spermatogenesezyklus) eine Erholung der Samenqualität bei einer Mehrheit der Männer erwartet werden darf [17]. Diese zu erwartende progressive Erholung der Samenqualität mit der Zeit hängt aber ganz deutlich vom Schweregrad und vom Inflammationszustand und somit vom zeitlichen Verlauf der Infektion ab [18]. Kritisch muss allerdings beurteilt werden, dass die durch die Infektion verursachten DNA-Schädigungen der Spermien einerseits zumindest vorübergehend die Fertilität des Betroffenen, aber, wenn es denn trotzdem zu einer Schwangerschaft kommt, eine Bedrohung für das Neugeborene bedeuten können [19].

## Langzeiteffekte durch COVID-19

Die in der Schweiz durchgeführte sog. LoCoMo, eine longitudinale Kohortenstudie, die insgesamt 501 Schweizer Militärzugehörige mit einem Altersdurchschnitt von 21 Jahren einschloss, zeigte in den untersuchten Gruppen, dass bei denjenigen, deren COVID-19-Erkrankung länger als 180 Tage zurücklag, im Vergleich mit der gesunden Gruppe (nie COVID-19) im Langzeitverlauf der BMI sowie das LDL- und Gesamtcholesterin höher und die Leistungsfähigkeit deutlich geringer waren [20].

## Einfluss durch Impfung

Als häufigste Nebenwirkung einer Impfung kann es zu Fieber kommen. Dieser potenzielle Einfluss wurde weiter oben bereits beleuchtet. Unter Anwendung der beiden mRNA-Impfstoffe von Pfizer-BioNTech und Moderna bezogen sich die Nebenwirkungen bei 15.785 Personen lediglich in 0.7 % (113) Fällen auf urologische Symptome [21]. Dabei kam es in 34 Fällen zu Miktionsstörungen, in 22 Fällen zu Hämaturie und in 41 Fällen zu Urininfektionen, wobei in rund 46 % ein männliches Geschlecht vorlag.

Beruhigender Weise konnte mehrfach gezeigt werden, dass es keinen negativen Einfluss auf die Samenparameter hatte und auch kein erhöhtes Risiko für eine erektile Dysfunktion [22, 23].

## Empfehlungen bei Fertilitätswunsch

Der Einfluss von Fieber, der Schweregrad der Erkrankung und der Erholungszustand sollten in Bezug auf eine angestrebte Reproduktion und bei Fertilitätswunsch berücksichtigt werden. Es sollte mindestens 3 Monate, tendenziell sogar etwas länger, auf eine (sei es natürliche oder artifizielle) Reproduktion nach dem Abklingen der Infektion gewartet werden. Eine Samenkryopräservation, z. B. bei Krebspatienten, scheint trotzdem möglich. Unter den pandemischen Umständen und unter Abwägung von Nutzen und potenziellen Risiken sollte Männern, welche eine Fertilität anstreben und die Kriterien für die Impfung erfüllen, diese nicht vorenthalten werden. COVID-19-infizierte Männer bedürfen im Hinblick auf eine angestrebte Fertilität eine enge Betreuung und Beratung [24].

## Testosteron und COVID-19

In einer Metaanalyse von 11 vorliegenden Studien, die Männer mit COVID-19 und entsprechende gesunde Kontrollgruppen verglichen, konnte gezeigt werden, dass Männer mit einem per se niedrigeren Testosteronlevel zu Beginn häufiger infiziert waren. Männer mit einem niedrigen Testosteronwert tragen ein 4‑ bis 5fach erhöhtes Risiko, im Verlauf der Infektion auf die Intensivstation verlegt zu werden oder gar zu versterben [15]. Wenn die Gesamttestosteronkonzentration < 100 ng/dl betrug, im Vergleich zu Testosteronwerten über 230 ng/dl, war das Mortalitätsrisiko signifikant höher („odds ratio“ 18,2, *p* = 0,006; [25, 26]). Die Erholung des Testosteronstoffwechsels benötigt im Vergleich zur Erholung der Samenqualität deutlich länger. So zeigte eine Studie erst nach 7 Monaten ein Erreichen von 87,6 % des Testosteronanstiegs im Vergleich zur Baseline zu Beginn der Infektion. Einzelne Studien berichteten aber auch noch längere Erholungszeiten mit bis zu 50 % anhaltend tiefen Testosteronwerten, welche die laborchemischen Kriterien eines Hypogonadismus erfüllen würden [27]. Die Rolle einer möglichen und frühzeitigen Testosteronergänzungstherapie ist zurzeit noch gänzlich ungeklärt [28]. Vorsicht ist bei diesem Gedanken der Tatsache geschuldet, dass COVID-19 mit einer Hyperkoagulabilität und eine Testosteronersatztherapie andererseits mit einem erhöhten Risiko einer Venenthrombose und einer Polyzythämie (HKT > 54 %) einhergehen kann.

## Erektile Dysfunktion (ED) und COVID-19

Bei einem Patienten, der sich 7 Monate nach einer COVID-19-Infektion einem chirurgischen Eingriff am Penis unterzog, konnte im Endothelium der Schwellkörper die Persistenz des SARS-CoV-2-Virus festgestellt werden [5]. Bei COVID-19, insbesondere mit schwerwiegenden Verläufen, konnte eine gravierende Schädigung des Endothels der Gefässe gezeigt werden. So ist es naheliegend, dass eine solche Vaskulopathie, welche mindestens mit einer mikrovaskulären Schädigung einhergeht, sich auch durch eine vaskulär bedingte bemerkbar machen kann [29]. Additiv können sich diesbezüglich auch noch, wie bereits beschrieben, hormonelle Einflüsse (möglicher Mangel an Testosteron) und auch psychischer Stress durch eine solche Infektion negativ bemerkbar machen. In Zahlen konnte eine Studie belegen, dass die Prävalenz einer ED bei Männern mit COVID-19 im Vergleich zu einer gesunden Kontrollgruppe signifikant höher war („odds ratio“ 5,66; [30]).

## Resümee

SARS-CoV‑2 ist ein höchst pathogener Virus, der eine schädigende Wirkung auf verschiedenste Art und Weise bewirken kann. Trotz der wachsenden Literatur kann der Einfluss der COVID-19-Pandemie in Bezug auf potenzielle Auswirkungen noch keineswegs überblickt werden. Unter andrem kann sich COVID-19 in Bezug auf die mentale Gesundheit (Depression und Angstzustände), die sexuelle Gesundheit (endotheliale Dysfunktion, erektile Dysfunktion) und die testikuläre Funktion (Orchitis, Samenqualität und Testosteron) bemerkbar machen. Es gibt noch viele offene Fragen, warum es bei Männern im Vergleich zu Frauen zu einem schwereren Krankheitsverlauf und einer höheren Sterblichkeit kommt. Männer weisen vergleichsweise mehr negative Komorbiditäten auf – auch das Vorliegen von Androgenrezeptoren, als potenzieller Mechanismus für einen Zelleintritt des Virus, scheint eine Rolle dafür zu spielen. Daher sollten COVID-19-Infizierte bei Bedarf sowohl in der akuten als auch in der Erholungsphase eine entsprechende Aufmerksamkeit erhalten.

**Keypoints:**

- Männer haben einen schwereren Verlauf und eine höhere Sterblichkeitsrate.
- Sexuelle Transmission erscheint eher unwahrscheinlich, kann aber nicht 100%ig ausgeschossen werden.
- Samenqualität unter COVID-19 wird deutlich schlechter, aber erholt sich wieder.
- Niedriger Testosteronwert resultiert in einem schwereren Krankheitsverlauf.
- COVID-19-Infektion kann zu einer (vorübergehenden) erektilen Dysfunktion führen.
- Impfung (mRNA-Vakzine) hat keinen negativen Einfluss auf die Samenqualität.

## Fazit für die Praxis

- Männer mit COVID-19 haben gegenüber Frauen ein höheres Risiko für einen schwereren Krankheitsverlauf (Intensivstation: „odds ratio“ 2,84) und eine höhere Mortalität („odds ratio“ 1,39).
- Das Risiko einer sexuellen Transmission des SARS-CoV‑2 nach Erholung in einer stabilen Partnerschaft scheint unwahrscheinlich.
- COVID-19 hat einen negativen (Kurzzeit‑)Effekt auf die Samenqualität, das Testosteron und die Erektionsfunktion – mögliche Langzeitkonsequenzen sind noch nicht hinreichend geklärt.
- Bis zu 1/4 von zuvor fertilen Männern zeigten unter COVID-19 und danach schwerste Spermatogenesestörungen (Oligo‑/Krypto‑/Azoospermie), abhängig von vorliegenden Komorbiditäten und dem Schweregrad der Infektion. Hingegen scheint eine Erholung nach wenigstens 3 Monaten wahrscheinlich.
- Aufgrund potenzieller infektbedingter DNA-Schäden im Erbgut benötigen Männer in Bezug auf eine angestrebte Fertilität, inklusive assistierter reproduktionsendokrinologischer Massnahmen und Kryopräservation, eine gute Beratung und einen engen Follow-up.
- Die verfügbaren mRNA-COVID-19-Impfungen scheinen weder Samenqualität noch Erektionsfunktion zu beeinflussen.
- Ein erniedrigtes Testosteronniveau scheint per se mit einem schwerwiegenderen COVID-19-Verlauf einherzugehen. Die potenzielle Rolle einer Androgendeprivationstherapie oder einer Testosteronsubstitutionstherapie müssen in diesem Zusammenhang noch geklärt werden.
- In der Erholung nach COVID-19 sollten Männer auch in Bezug auf Samenqualität und Testosteronabnormalitäten kontrolliert werden.

## References

[1] Moeling K (2021). Within-host and between-host evolution in SARS-CoV-2-new variant’s source. Viruses.

[2] Li Xuhua QG, Peng W (2020). Early transmission dynamics in Wuhan, China, of novel coronavirus-infected pneumonia. N Engl J Med.

[3] Pradhan A, Olsson P-E (2020). Sex differences in severity and mortaliy form COVID-19: are males more vulnerable ?. Biol Sex Differ.

[4] Nassau DE, Best JC, Kresch E, Gonzalez DC, Khodamoradi K, Ramasamy R (2022). Impact of the SARS-CoV-2 virus on male repdoductive health. BJU Int.

[5] Aitken RJ (2021) COVID-19 and human spermatozoa-Potential risks for infertility and sexual transmission? Andrology 9(1):48–52 10.1111/andr.12859 PMC7404878 32649023

[6] Li H, Liu L, Zhang D, Xu J, Dai H, Tang N, Su X, Cao B (2020). SARS-CoV-2 and viral sepsis : observations and hypotheses. Lancet.

[7] Villiamson EJ, Walker AJ, Bhaskaran K (2020). Factors associated with COVID-19-related death using OpenSAFELY. Nature.

[8] Yang J, Wang W, Chen Z (2020). A Vaccine targeting the RBD of the Sprotein of SARS-CoV-2 induces protecitve immunity. Nature.

[9] Hoffmann M, Kleine-Weber H, Schroeder S (2020). SARS-CoV-2 Cell Entry Depends on ACE2 and TMPRSS2 and is blocked by a clinically proven protease Inhibitor. Cell.

[10] Buonacquisto A, Conflitti AC, Pallotti F (2022). COVID-19 Severity and Androgen Receptor Polymorphism. Biol (basel).

[11] Ediz C, Tavukcu HH, Akan S, Kizilkan YE, Alcin A, Oz K, Yilmaz O (2021). Is there any association of COVID-19 with testicular pain and epididymo-orchitis ?. Int J Clin Pract.

[12] Abdelhamid MHM, Fellah AA (2023). Elmarghani A Msellati IAA. An Assessment of Men Semen Alterations in SARS-CoV-2: Is Fever the Principa Concern ?. Reprod Sci.

[13] Koc E, Keseroglu BB (2021). Does COVID-19 Worsen the semen parameters ? Early results of a Tertiary Healthcare Center. Urol Int.

[14] Gacci M, Coppi M, Baldi E (2021). Semen impairment and occurrence of SARS-CoV-2 virus in semen after recovery form COVID-19. Hum Reprod.

[15] Coroan G, Vena W, Pizzocaro A (2022). Andrological effects of SARS-Cov-2 infection : a systematic review and meta-analysis. J Endrocrinol Invest.

[16] Xie Y, Mirzaei M, Kahrizi MS, Shabestrari AM, Riahi SM, Farsimadan M, Roviello G (2022). SARS-CoV-2 effects on sperm parameters : a meta-analysis study. J Assist Reprod Genet.

[17] Paoli D, Pallotti F, Anzuini A et al (2022) Male reproductive health after 3 month from SARS-CoV‑2 infection : a multicentric study. J Endrocrinol Invest 46(1):89–1001 10.1007/s40618-022-01887-3 PMC9362397 35943723

[18] Hajizadeh Maleki B, Tartibian B (2021). COVID-19 and male reproductive function : a prospective, longitudianl cohort study. Reproduction.

[19] Aitken RJ, Bakos HW (2021). Shoulrd we be measuring DNA damage in human spermatozoa? New light on an old question. Hum Reprod.

[20] Deuel JW, Lauria E, Lovey T, Zweifel S, Meier MI, Züst R, Gültekin N, Stettbacher A, Schlagenhauf P (2022). Persistence, prevalence, and polymorphism of sequelae after COVID-19 in unavaccinated, young adults oft he Swiss Armed Forces: a longitudinal, cohort study (LoCoMo). Lancet Infect Dis.

[21] Zhao H, Sounders C, Carmel M, Anger JT (2021). Low Rates of Urologic Side Effects following Coronavirus disease vaccination : an analysis of the food and drug adminstration vaccine adverse event reporting system. Urology.

[22] Safrai M, Herzberg S, Imbar T, Reubinoff B, Dior U, Ben-Meir A (2022). The BNT162b2 mRNA Covid-19 vaccine does not impair sperm parameters. Reprod Biomed Online.

[23] Gonzalez DC, Nassau DE, Khodamoradi K, Ibrahim E, Blachman-Braun R, Ory J, Ramasamy R (2021). Sperm Parameters before and after COVID-19 mRNA vaccination. JAMA.

[24] (2021) Joint Statement SMRU and SSMR. https://ssmr.org/news/statement-from-ssmr-about-covid-19-vaccine.aspx. Zugegriffen: 22.08.2022

[25] Kadihasanoglu M, Aktas S, Yardimci E, Aral H, Kadioglu A (2021). SARS-CoV-2 Peneumonia affets male reproductive hormone levels : a prospective , cohort study. J Sex Med.

[26] Salonia A, Pontillo M, Capogrossa P (2021). Severely low testosterone in males with COVID-19: A case-control study. Andrology.

[27] Salonia A, Pontillo M, Capogrossa P (2022). Testosterone in males with COVID-19: A 7-month cohort study. Andrology.

[28] Rambhatla A, Bronkema CJ, Corsi N (2021). COVID-19 infection in men on testosterone replacement therapy. J Sex Med.

[29] Sansone A, Mollaioli D, Giocca G, Limoncin E, Colonnello E, Vena W, Jannini EA (2021). Addressing male sexual and reproductive health in the wake of COVID-19. Outbreak J Endrocrinol Invest.

[30] Sansone A, Mollaioli D, Ciocca G, Colonnello E, Limoncin E, Balercia G, Jannini EA (2021). “Mask up to keep it up”: Preliminary evidence of the association between erectile dysfunction and COVID-19. Andrology.

